# Re-examining cephalosporin activity against methicillin-susceptible *Staphylococcus aureus* among clinical isolates from southern Taiwan

**DOI:** 10.1093/jacamr/dlag029

**Published:** 2026-03-05

**Authors:** Yin-Ting Lin, Shu-Fang Kuo, Chun-Chih Chien, Chien-Hui Hung, Hui-Yen Ming, Tsung-Yu Huang, Chen-Hsiang Lee

**Affiliations:** Division of Infectious Diseases, Department of Internal Medicine, Chiayi Chang Gung Memorial Hospital, Chiayi, Taiwan; Microbiology Treatment and Research Center, Chiayi Chang Gung Memorial Hospital, Chiayi, Taiwan; Microbiology Treatment and Research Center, Chiayi Chang Gung Memorial Hospital, Chiayi, Taiwan; Departments of Laboratory Medicine, Chiayi Chang Gung Memorial Hospital, Chiayi, Taiwan; Department of Medical Biotechnology and Laboratory Sciences, College of Medicine, Chang Gung University, Taoyuan, Taiwan; Department of Laboratory Medicine, Kaohsiung Chang Gung Memorial Hospital, Kaohsiung, Taiwan; Division of Infectious Diseases, Department of Internal Medicine, Chiayi Chang Gung Memorial Hospital, Chiayi, Taiwan; Graduate Institute of Clinical Medical Sciences, College of Medicine, Chang-Gung University, Taoyuan, Taiwan; Division of Infectious Diseases, Department of Internal Medicine, Chiayi Chang Gung Memorial Hospital, Chiayi, Taiwan; Division of Infectious Diseases, Department of Internal Medicine, Chiayi Chang Gung Memorial Hospital, Chiayi, Taiwan; Microbiology Treatment and Research Center, Chiayi Chang Gung Memorial Hospital, Chiayi, Taiwan; College of Medicine, Chang Gung University, Taoyuan, Taiwan; Division of Infectious Diseases, Department of Internal Medicine, Chiayi Chang Gung Memorial Hospital, Chiayi, Taiwan; College of Medicine, Chang Gung University, Taoyuan, Taiwan; Division of Infectious Diseases, Department of Internal Medicine, Kaohsiung Chang Gung Memorial Hospital, Kaohsiung, Taiwan

## Abstract

**Background:**

Cephalosporin susceptibility in methicillin-susceptible *Staphylococcus aureus* (MSSA) is typically inferred from oxacillin or cefoxitin results; however, the reliability of this surrogate approach remains uncertain. This study aimed to evaluate MSSA susceptibility to first- through fifth-generation cephalosporins and assess the predictive value of oxacillin minimum inhibitory concentrations (MICs) for identifying non-susceptibility.

**Methods:**

A total of 514 MSSA bloodstream isolates were collected from two hospitals in Taiwan. MICs for oxacillin and various cephalosporins were determined using broth microdilution. Receiver operating characteristic (ROC) curve analysis was conducted to evaluate the predictive performance of oxacillin MICs. Multilocus sequence typing (MLST) was performed on isolates exhibiting ceftaroline resistance or the susceptible-dose dependent (SDD) phenotype to examine their genetic diversity.

**Results:**

Non-susceptibility rates ranged from 4.9% to 6.2% for cefazolin, cefuroxime, ceftriaxone, and cefepime. For ceftaroline, while resistance was uncommon (1.4%), a notable proportion of isolates (9.7%) exhibited the SDD phenotype. An oxacillin MIC ≥0.25 mg/L predicted non-susceptibility to first- through fourth-generation cephalosporins with high accuracy (area under the curve [AUC] 0.827–0.875; negative predictive value [NPV] ≥ 98.7%) but was less predictive for ceftaroline (AUC 0.614). Among 44 isolates with elevated ceftaroline MICs, MLST identified 36 distinct sequence types, including 25 novel ones, indicating substantial genetic diversity.

**Conclusions:**

Cephalosporin non-susceptibility can occur among MSSA isolates. Although elevated oxacillin MICs were associated with higher MICs for several cephalosporins, this relationship was less predictive for ceftaroline. These findings highlight microbiological variability in MSSA cephalosporin susceptibility and may inform situations where direct MIC testing provides additional clarity.

## Introduction

*Staphylococcus aureus* is a major pathogen responsible for both community-acquired and healthcare-associated infections, contributing significantly to global morbidity and mortality, particularly in cases of bacteraemia.^1,2^ Although the incidence of methicillin-resistant *S. aureus* (MRSA) bloodstream infections (BSIs) has declined in recent years, several surveillance studies have reported a concurrent increase in methicillin-susceptible *S. aureus* (MSSA) BSIs, underscoring its growing clinical impact.^3,4^

Effective treatment of MSSA relies primarily on β-lactam antibiotics, with anti-staphylococcal penicillins (ASPs) such as oxacillin historically regarded as standard therapy, although cefazolin is now preferred in many centers.^5–7^ Retrospective studies have shown that cefazolin is as effective as ASPs for treating MSSA bacteraemia.^8,9^ Its convenient dosing, favourable pharmacokinetics, lower cost, and reduced risk of adverse effects have contributed to its widespread clinical adoption.^8–10^ In addition, second- and third-generation cephalosporins, such as cefuroxime and ceftriaxone, exhibit activity against MSSA and are occasionally used as alternative options in selected clinical contexts, particularly when broader Gram-negative coverage is desired.

In 2012, the Clinical and Laboratory Standards Institute (CLSI) eliminated minimum inhibitory concentration (MIC) breakpoints for most β-lactams, retaining only those for penicillin, oxacillin, cefoxitin, and ceftaroline due to concerns about reliability and clinical relevance.^11^ This revision aimed to reduce the risk of inaccurate susceptibility reporting, particularly for MRSA, and to simplify laboratory workflows. Although oxacillin or cefoxitin MIC testing is commonly used as a surrogate for cephalosporin susceptibility in MSSA, the reliability of this approach remains uncertain. A US surveillance study of 4016 MSSA isolates collected between 2008 and 2010 reported a 4.0% non-susceptibility rate to ceftriaxone.^12^ Moreover, another study revealed that cefoxitin produced an unacceptably high rate of major errors when predicting susceptibility to cefazolin, ceftaroline, ceftriaxone, and nafcillin, raising questions about its utility as a surrogate indicator.^13^ These findings highlight the possibility of elevated MICs being overlooked when inferred susceptibility is used.

Despite these concerns, comprehensive evaluations of cephalosporin susceptibility in MSSA remain limited. This study aimed to assess the susceptibility of MSSA to first- through fifth-generation cephalosporins and to evaluate the reliability of oxacillin and cefoxitin MICs as surrogate markers. Given concerns regarding the poor accuracy of cefoxitin-based predictions,^13^ we focused on the predictive value of oxacillin MICs for identifying cephalosporin non-susceptibility. Establishing this relationship may improve susceptibility inference and support antimicrobial decision-making for MSSA infections.

## Methods

### Study population and setting

This study was conducted at two tertiary care hospitals in southern Taiwan: Chiayi Chang Gung Memorial Hospital (CCGMH; 1300 beds) and Kaohsiung Chang Gung Memorial Hospital (KCGMH; 2600 beds). *S. aureus* isolates were obtained from the bacterial strain banks of both hospitals, originating from blood cultures collected between January 2015 and December 2024. The study protocol was approved by the Institutional Review Board, with a waiver of informed consent (IRB Nos.: 202300428B1 and 202400473B1).

### Microbiological studies

Bacterial identification and antimicrobial susceptibility testing were conducted by the clinical microbiology laboratories at each site between 2015 and 2024. At KCGMH, identification was performed using matrix-assisted laser desorption/ionization time-of-flight mass spectrometry (MALDI-TOF MS; Bruker Daltonik, Bremen, Germany) throughout the study period. MICs were determined using the BD Phoenix^™^ automated system (BD Biosciences), initially based on the M100 system and later upgraded to the M50 system in January 2020. At CCGMH, conventional biochemical methods were used for identification until December 2018, after which MALDI-TOF MS was implemented. Susceptibility testing transitioned from the disk diffusion method to MIC testing using the BD Phoenix^™^ M50 system in June 2018. MSSA was defined as *S. aureus* isolates susceptible to both oxacillin and cefoxitin. All laboratory procedures followed CLSI guidelines.^14^

### Antimicrobial susceptibility testing

The broth microdilution method was performed in accordance with CLSI guidelines.^15^ Antibiotics were serially diluted twofold in 96-well microtiter plates containing Mueller–Hinton broth (Merck Millipore, Burlington, MA, USA). A final inoculum of approximately 5 × 10⁵ colony-forming units per millilitre (CFU/mL) was prepared for each well. Plates were incubated at 35°C; oxacillin MIC testing was incubated for 24 h to ensure detection of heteroresistance, whereas incubation for all cephalosporins was 18–20 h, consistent with CLSI recommendations for β-lactam agents. The MIC was defined as the lowest antibiotic concentration that completely inhibited visible bacterial growth. *S. aureus* ATCC 29213 was used as the quality control strain, and all MIC values were within the CLSI-specified acceptable ranges (Table S1, available as Supplementary data at *JAC-AMR* Online).

The tested antibiotics included oxacillin, cefazolin, cefuroxime, ceftriaxone, cefepime, and ceftaroline. MIC breakpoints for oxacillin and ceftaroline were interpreted according to CLSI M100, 34th edition.^14^ For cefazolin, cefuroxime, ceftriaxone, and cefepime, CLSI M100-S22 breakpoints were applied, as no updated criteria are currently available.^16^ All breakpoint values are listed in Table S2.

### Multilocus sequence typing of ceftaroline-resistant or susceptible-dose dependent MSSA isolates

An unexpected distribution of ceftaroline MICs prompted further molecular investigation. MSSA isolates classified as ceftaroline-resistant or susceptible-dose dependent (SDD) underwent multilocus sequence typing (MLST). Seven housekeeping genes (*arcC*, *aroE*, *glpF*, *gmk*, *pta*, *tpi*, and *yqiL*) were amplified according to established protocols.^17^ Polymerase chain reaction (PCR) and sequencing were performed by TRI-I Biotech (Taipei, Taiwan). Sequence types (STs) were assigned using the *S. aureus* MLST database (https://pubmlst.org/saureus/), and novel allelic profiles were submitted for assignment of new STs. Clonal relatedness was analysed using PHYLOViZ (http://www.phyloviz.net).

### Statistical analysis

Statistical analyses were performed using IBM SPSS Statistics version 23 (IBM Corp., Armonk, NY, USA). To evaluate temporal trends, the study period was stratified into two 5-year intervals (2015–2019 and 2020–2024), allowing balanced comparison across the decade-long cohort. Categorical variables were compared using the chi-square test or Fisher’s exact test. Receiver operating characteristic (ROC) curves were generated, and the area under the curve (AUC) with 95% confidence intervals (CIs) was calculated to evaluate the ability of oxacillin MIC to predict cephalosporin non-susceptibility. Optimal cut-off values were determined by maximizing the Youden index. Sensitivity, specificity, positive predictive value (PPV), and negative predictive value (NPV) were calculated for each antimicrobial agent.

## Results

### Antimicrobial susceptibility profiles

During the study period, a total of 884 non-duplicate *S. aureus* isolates were recovered from BSIs. MSSA isolates were defined as susceptible to both cefoxitin and oxacillin, based on routine susceptibility testing performed in our laboratories (disk diffusion or the BD Phoenix^™^ system) in accordance with CLSI recommendations. This approach yielded 514 MSSA isolates, comprising 346 isolates from CCGMH collected between 2015 and 2024, and 168 isolates from KCGMH collected between 2020 and 2024. These isolates were subsequently subjected to study-specific broth microdilution testing. The antimicrobial susceptibility results for the tested cephalosporins are summarized in Table 1. The non-susceptibility rates, including intermediate and resistant categories, were 4.9% for cefazolin, 5.7% for cefuroxime, 5.0% for ceftriaxone, and 6.2% for cefepime. For ceftaroline, resistance was observed in 1.4% of isolates, while 9.7% were classified as SDD. The detailed MIC distributions for all tested cephalosporins are presented in Figure S1.

**Table 1.** Cephalosporins susceptibility proﬁle of methicillin-susceptible *Staphylococcus aureus* (*n* = 514)

Antimicrobial agents	Susceptibility (%)		
	*S*	SDD/I	*R*
Cefazolin	95.1	1.2	3.7
Cefuroxime	94.3	1.4	4.3
Ceftriaxone	95.0	2.5	2.5
Cefepime	93.8	2.5	3.7
Ceftaroline	88.9	9.7*	1.4
Oxacillin	100	—	0

I, intermediate; R, resistant; S, susceptible; susceptible-dose dependent.

^*^Susceptible-dose dependent for ceftaroline.

### Temporal trends in cephalosporin non-susceptibility

To evaluate temporal trends and regional consistency, MSSA isolates were stratified by hospital and collection period (Figure S2). At CCGMH, where surveillance data spanned the entire study period, non-susceptibility rates declined significantly between the earlier (2015–2019, *n* = 186) and later (2020–2024, *n* = 160) intervals. Specifically, non-susceptibility decreased from 8.6% to 2.5% for cefazolin, from 11.3% to 1.9% for cefuroxime, from 9.7% to 1.9% for ceftriaxone, and from 11.3% to 2.5% for cefepime (all *P* < 0.01). In contrast, ceftaroline resistance rate remained consistently low, with no statistically significant difference observed between the two periods (2.2% versus 0.6%).

During the concurrent period (2020–2024), susceptibility profiles were compared between CCGMH and KCGMH (*n* = 168). Non-susceptibility rates were highly comparable between the two institutions, with no statistically significant differences observed for cefazolin (2.5% versus 3.0%), cefuroxime (1.9% versus 3.0%), ceftriaxone (1.9% versus 3.0%), cefepime (2.5% versus 4.2%), and ceftaroline (0.6% versus 1.1%).

### Predictive performance of oxacillin MIC for cephalosporin non-susceptibility

**Figure 1.**
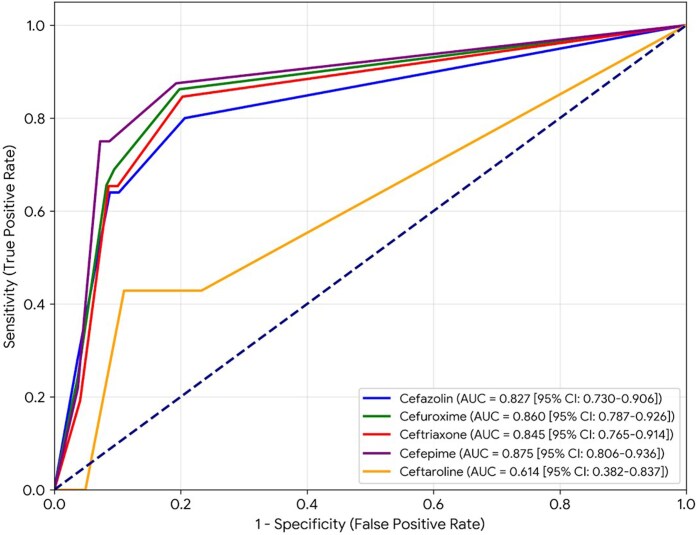
ROC curves evaluating the predictive value of oxacillin minimum inhibitory concentration for cephalosporin non-susceptibility in methicillin-susceptible *Staphylococcus aureus* isolates.

Receiver operating characteristic (ROC) curve analysis was performed to assess the ability of oxacillin MICs to predict non-susceptibility to individual cephalosporins (Figure 1). The area under the curve (AUC) values with 95% confidence intervals (CIs) were as follows: cefazolin, 0.827 (0.730–0.906); cefuroxime, 0.860 (0.787–0.926); ceftriaxone, 0.845 (0.765–0.914); cefepime, 0.875 (0.806–0.936); and ceftaroline, 0.614 (0.382–0.837). All cephalosporins except ceftaroline demonstrated good discriminatory performance, with AUCs exceeding 0.8.

As summarized in Table 2, an oxacillin MIC cut-off of ≥0.25 mg/L provided optimal discrimination for predicting cephalosporin non-susceptibility in MSSA. This threshold demonstrated good sensitivity (80.0%–87.5%) and specificity (79.3%–80.7%) for cefazolin, cefuroxime, ceftriaxone, and cefepime, along with consistently high NPVs (≥98.7%). However, ceftaroline exhibited poorer predictive performance, with a sensitivity of 71.4%, specificity of 79.3%, and an NPV of 99.5%. For first- to fourth-generation cephalosporins, non-susceptibility rates remained low (1.0%–1.3%) when the oxacillin MIC was <0.25 mg/L but increased markedly (16.5%–23.1%) at MICs ≥0.25 mg/L. In contrast, the increase in ceftaroline resistance was less pronounced, rising from 0.5% at oxacillin MIC <0.25 mg/L to 4.1% at oxacillin MICs ≥0.25 mg/L (Figure S3). The correlation patterns between oxacillin and cephalosporin MICs are further visualized in Figure S4.

**Table 2.** Optimal oxacillin minimum inhibitory concentration thresholds and diagnostic performance for predicting cephalosporin non-susceptibility in methicillin-susceptible *Staphylococcus aureus*

Antibiotic	Optimal threshold (oxacillin MIC, mg/L)	Youden Index	Sensitivity (%)	Specificity (%)	PPV (%)	NPV (%)
Cefazolin	≥0.25	0.593	80.0	79.3	16.5	98.7
Cefuroxime	≥0.25	0.664	86.2	80.2	20.7	99.0
Ceftriaxone	≥0.25	0.643	84.6	79.7	18.2	99.0
Cefepime	≥0.25	0.682	87.5	80.7	23.1	99.0
Ceftaroline	≥0.25	0.507	71.4	79.3	4.1	99.5

MIC, minimum inhibitory concentration; NPV, negative predictive values; PPV, positive predictive value.

### Genetic diversity among ceftaroline-resistant or susceptible-dose dependent isolates

**Figure 2.**
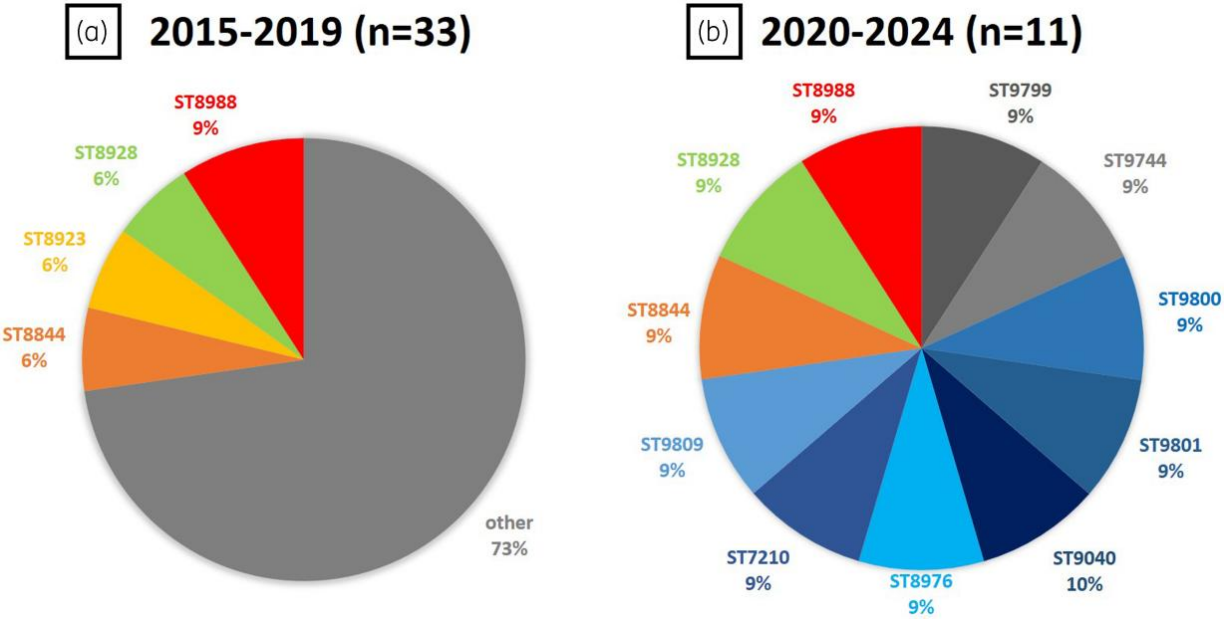
Distribution of sequence types (STs) among ceftaroline-resistant or susceptible-dose dependent methicillin-susceptible *Staphylococcus aureus* isolates from CCGMH during 2015–2024. (a) The 2015–2019 group (*n* = 33) showed some major STs. (b) The 2020–2024 group (*n* = 11) demonstrated genetic heterogeneity, with no repeated STs.

Given the high prevalence of the ceftaroline SDD phenotype (9.7%) and the presence of resistance (1.4%), MLST was performed on 44 isolates with ceftaroline-resistant and SDD. MSSA isolates from CCGMH to investigate their genetic diversity. A total of 36 distinct sequence types (STs) were identified, including 25 novel STs (ST9785–ST9809), which have been submitted to the *S. aureus* MLST database. The distribution of major STs is presented in Figure 2. Among the 33 isolates collected during 2015–2019, several STs were shared among multiple isolates, including ST8988 (*n* = 3; 9%), ST8928 (*n* = 2; 6%), ST8844 (*n* = 2; 6%), and ST8923 (*n* = 2; 6%). The remaining 24 isolates each possessed a unique ST. In contrast, all 11 isolates from the 2020–2024 period exhibited complete genetic heterogeneity, with no repeated STs identified.

**Figure 3.**
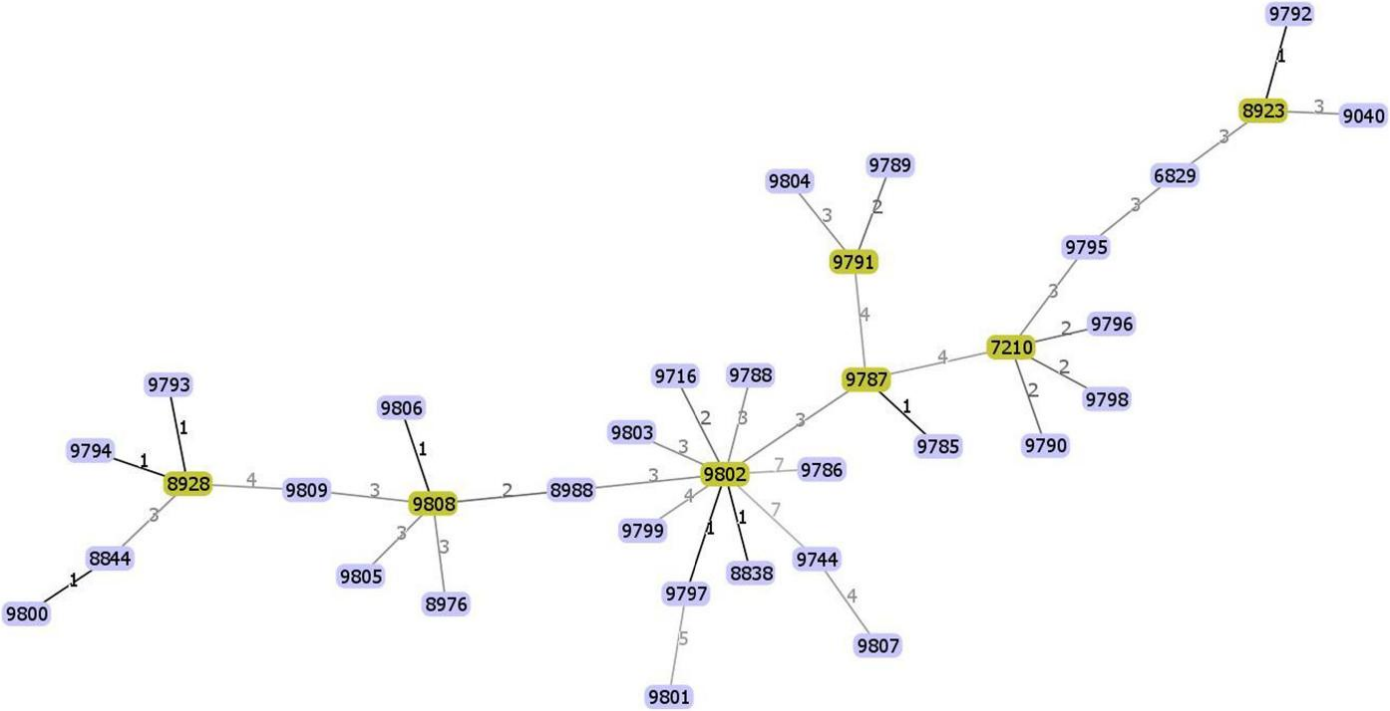
Minimum spanning tree based on multilocus sequence typing of ceftaroline-resistant or susceptible-dose dependent methicillin-susceptible *Staphylococcus aureus* isolates from CCGMH during 2015–2024. Each oval represents a sequence type (ST). Yellow nodes denote founder STs of clonal complexes. Edge labels indicate the number of allelic differences between connected STs; single-locus variants share 6 out of 7 alleles with their founder, while others are multi-locus variants.

A comprehensive list of STs and their corresponding clonal complexes (CCs) is provided in Table S3. Clonal relatedness was analysed using the PHYLOViZ platform, which groups STs sharing at least five of the seven housekeeping gene alleles into the same CC. Seven distinct CCs were identified, with the following founder STs: ST7210, ST8923, ST8928, ST9787, ST9791, ST9802, and ST9808 (Figure 3). For instance, the genetic distance between ST9785 and ST9787 was 1, indicating minimal evolutionary divergence and a close genetic relationship. In contrast, STs with greater allelic differences exhibited more distant genetic relationships, reflecting increased evolutionary divergence within the dataset.

## Discussion

In clinical practice, the susceptibility of *S. aureus* to cephalosporins is typically inferred from oxacillin or cefoxitin results. This approach has been endorsed by the CLSI since 1991 and incorporated into the M100 guidelines accordingly.^11^ While MIC breakpoints for several β-lactams were initially retained, most were removed in 2012, with only penicillin, oxacillin, cefoxitin, and ceftaroline remaining.^11,13,18^ The main rationale for this revision was to prevent misclassification of MRSA, as certain MRSA strains may test falsely susceptible to cephalosporins or carbapenems by *in vitro* testing.^11,18^ However, the validity of applying this inferred susceptibility framework to MSSA has not been thoroughly validated. In our study, we evaluated 514 MSSA bloodstream isolates, each initially identified as oxacillin-susceptible using the broth microdilution method. The non-susceptibility rates for cefazolin, cefuroxime, ceftriaxone, and cefepime ranged from 4.9% to 6.2%. Although the ceftaroline non-susceptible rate was relatively low (1.4%), a notable proportion of isolates (9.7%) fell into the SDD category. Among isolates collected between 2015 and 2019 in CCGMH, the non-susceptibility rates were even higher, ranging from 8.6% to 11.3% for cefazolin to cefepime, whereas ceftaroline resistance remained low at 2.4%. These findings indicate that inferred cephalosporin susceptibility may not always reflect actual MIC distributions in MSSA and highlight the need for further evaluation of circumstances in which direct MIC testing might provide additional clarity.

The variability in cephalosporin MICs among MSSA isolates may reflect differences in the binding affinities of these agents for penicillin-binding proteins (PBPs), which are essential for bacterial cell wall synthesis. Oxacillin primarily targets PBP1 and PBP2, whereas cefoxitin shows greater affinity for PBP4.^19,20^ In comparison, cephalosporins exhibit distinct PBP-binding profiles: cefazolin mainly binds PBP1 and PBP2; ceftriaxone and cefepime target PBP2 and PBP3; and ceftaroline interacts with multiple PBPs, including PBP2a, thereby retaining activity against MRSA.^21,22^ These differences in PBP-binding patterns likely contribute to the discrepancies observed between oxacillin or cefoxitin susceptibility and cephalosporin MICs among MSSA isolates.^22^

Cefazolin remains a mainstay of therapy for MSSA infections; however, the cefazolin inoculum effect (CzIE), a pronounced rise in MIC under high-inoculum conditions, has been proposed as a potential concern. Although robust clinical evidence linking CzIE to patient outcomes is still limited, multiple *in vivo* animal studies suggest that high bacterial burdens can compromise cefazolin activity.^23,24^ In prior work, CzIE-positive isolates were significantly more likely to exhibit standard-inoculum cefazolin MICs of 1.0 mg/L (29.8% versus 3.2% among CzIE-negative isolates), a value that remains within the susceptible range yet is relatively elevated.^25^ While CzIE testing was beyond the scope of this study, its possible impact warrants consideration when interpreting cefazolin MIC distributions. Attention has likewise shifted towards ceftriaxone, whose widespread use largely reflects once-daily dosing, absence of renal adjustment, and lower cost.^26^ However, clinical studies indicate that ceftriaxone is less effective than cefazolin or oxacillin for invasive MSSA infections,^27^ and pharmacokinetic/pharmacodynamic (PK/PD) analyses indicate that once-daily 2 g dosing may not reliably achieve target T > MIC or bactericidal exposures when the ceftriaxone MIC is ≥4 mg/L.^28,29^ Surveillance data from the SENTRY Programme (2011–2012), encompassing 14 335 MSSA isolates, showed that ceftriaxone resistance (MIC ≥8 mg/L) increased in parallel with oxacillin MICs—1.6%, 3.9%, 17.5%, and 48.7% at oxacillin MICs of ≤0.25, 0.5, 1, and 2 mg/L, respectively.^30^ Consistent with these findings, our study showed ceftriaxone non-susceptibility in 2.0% of isolates with oxacillin MIC ≤0.25 mg/L, 0% at 0.5 mg/L, 35.3% at 1 mg/L, and 20.0% at 2 mg/L. Taken together, our results indicate that cephalosporin non-susceptibility can occur even among isolates classified as oxacillin-susceptible by standard criteria. While robust clinical outcome data remain lacking, elevated MICs may serve as indirect indicators of phenomena such as the CzIE or the pharmacodynamic limitations of ceftriaxone at standard dosing that could theoretically influence drug performance in high-burden infections. The absence of routine cephalosporin MIC testing may therefore represent an important gap. In certain contexts, direct MIC testing may offer additional clarity beyond inferred results, particularly when oxacillin MICs are relatively elevated.

Because routine cephalosporin MIC testing is uncommon in many clinical laboratories, we examined whether oxacillin MIC values could serve as predictors of cephalosporin non-susceptibility. An oxacillin MIC threshold of ≥0.25 mg/L was identified for all tested cephalosporins, demonstrating strong predictive performance specifically for first- through fourth-generation agents, with AUCs ranging from 0.827 to 0.875 and NPVs of approximately 99%. These findings suggest that low oxacillin MICs may provide reassurance when applying inferred susceptibility. In contrast, isolates with relatively higher oxacillin MICs should not be considered non-susceptible, but may warrant closer review or, in selected clinical scenarios, consideration of direct MIC testing when precise cephalosporin activity is important. According to current CLSI guidelines, ceftaroline susceptibility in MSSA may be inferred from oxacillin or cefoxitin results, with direct testing reserved for MRSA.^14^ In our cohort, although the proportion of ceftaroline-resistant isolates was low (1.4%), oxacillin MIC demonstrated limited predictive accuracy for ceftaroline non-susceptibility (AUC 0.614). Notably, 9.7% of isolates fell into the SDD category, indicating that while these isolates remain potentially treatable, higher-dose regimens (e.g. 600 mg every 8 h) are often required to achieve PK/PD targets. Taken together, these observations suggest that direct ceftaroline MIC testing may provide additional clarity in situations where ceftaroline is being considered for the treatment and therapeutic decisions require greater certainty. The clinical relevance of SDD categories may therefore warrant consideration for both MSSA and MRSA, even though routine ceftaroline MIC testing is currently recommended by CLSI only for MRSA.^14^

In international surveillance programmes such as SENTRY and the Assessing Worldwide Antimicrobial Resistance Evaluation initiative, all MSSA isolates tested to date have demonstrated ceftaroline MICs within the susceptible range (≤1 mg/L). This contrasts with the emergence of SDD (9.7%) and resistant (1.4%) phenotypes observed in our cohort.^31,32^ The discrepancy suggests that local epidemiological factors may differ substantially from global trends. MLST is a valuable tool for elucidating the clonal lineages and molecular epidemiology of *S. aureus*, providing insights that inform infection control and prevention strategies. In Taiwan, MRSA infections have long posed a public health challenge, and the clonal spread of specific MRSA strains has been well documented.^33,34^ In contrast, studies focusing on MSSA remain limited.^35,36^ We performed MLST on 44 ceftaroline-resistant and SDD *S. aureus* isolates and identified 36 distinct STs, including 25 newly assigned STs not previously reported in Taiwan.^35,36^ These lineages also differ from globally prevalent STs,^37^ suggesting substantial genetic diversity. This finding indicates that the elevated ceftaroline MICs observed among MSSA in our study are unlikely to be driven by clonal expansion. Rather, it may reflect independent acquisition events or selective pressures acting within a genetically diverse population. This may partly explain why ceftaroline MIC distributions in our study differ from those observed in international surveillance programs,^31,32^ although the precise causes remain uncertain. Notably, we observed a gradual increase in ST diversity among more recent isolates (2020–2024); however, ceftaroline resistance did not increase in parallel with this diversification. Since ceftaroline was incorporated into Taiwan’s National Health Insurance system in February 2019, it remains unclear whether this policy change has contributed to enhanced selective pressure and subsequent diversification.

This study has several limitations. First, MSSA isolates were collected from only two institutions in southern Taiwan. While CCGMH contributed data spanning the entire 10-year period, isolates from KCGMH were available only from 2020 to 2024. Although recent data demonstrate high concordance in susceptibility profiles between the two hospitals, this temporal imbalance precludes a direct historical comparison between centres and limits our ability to determine whether the elevated non-susceptibility rates observed at CCGMH during 2015–2019 were present regionally. Second, the specific drivers and molecular lineages underlying the elevated non-susceptibility rates observed earlier (2015–2019) at CCGMH remain unclear, and MLST was performed only on 44 ceftaroline-resistant and SDD isolates from CCGMH. As a result, the generalizability of these findings to other regions is limited without further molecular and epidemiological investigation. As noted above, ceftaroline was incorporated into Taiwan’s National Health Insurance system in February 2019. Paradoxically, non-susceptibility rates for all tested cephalosporins declined after this policy change, suggesting that increased ceftaroline use is unlikely to have driven the resistance patterns observed prior to its widespread availability. Whether these temporal shifts reflect broader epidemiological changes, such as the replacement of specific high-MIC clones or the global decline in MRSA prevalence reported in recent years, remains uncertain.^3,4^ Moreover, genotypic testing for *mecA* and *mecC* was not performed, which could have provided additional confirmation of methicillin susceptibility and ensured the exclusion of oxacillin-susceptible *mecA*-positive MRSA (OS-MRSA). However, in our study, the classification strategy followed CLSI-recommended clinical microbiology laboratory workflows for MSSA screening and applied an even more conservative definition by requiring susceptibility to both oxacillin and cefoxitin. Additionally, whole-genome sequencing was not conducted. Consequently, the specific molecular mechanisms driving elevated ceftaroline MICs, including single-nucleotide polymorphisms or mutations in genes encoding PBPs and other resistance-associated determinants, could not be definitively characterized in the current study. Finally, we acknowledge that there is no strong evidence linking cephalosporin MIC patterns or minor elevations in cephalosporin MICs to clinical outcomes in MSSA infections. Accordingly, the clinical significance of MIC discordance remains uncertain, and future studies correlating susceptibility results with patient outcomes will be essential to determine the practical implications of these observations.

Overall, our findings demonstrate that cephalosporin non-susceptibility occurs among MSSA isolates. Although elevations in oxacillin MIC were associated with higher MICs for several cephalosporins, this relationship was less predictive for ceftaroline. Specifically, while ceftaroline resistance remains uncommon, the SDD phenotype is not rare and is frequently not identified by surrogate testing. These observations highlight microbiological variability in MSSA cephalosporin susceptibility and suggest that direct MIC testing may provide additional clinical clarity in selected situations.

## Supplementary Material

dlag029_Supplementary_Data

## Supplementary data

Figures S1 to S4 and Tables S1 to S3 are available as Supplementary data at *JAC-AMR* Online.

## References

[1] El Atrouni WI, Knoll BM, Lahr BD et al Temporal trends in the incidence of *Staphylococcus aureus* bacteremia in Olmsted County, Minnesota, 1998 to 2005: a population-based study. Clin Infect Dis 2009; 49: e130–8. 10.1086/648442 19916797 PMC3050712

[2] Goto M, Al-Hasan MN. Overall burden of bloodstream infection and nosocomial bloodstream infection in North America and Europe. Clin Microbiol Infect 2013; 19: 501–9. 10.1111/1469-0691.12195 23473333

[3] Renggli L, Gasser M, Buetti N et al Increase in methicillin-susceptible *Staphylococcus aureus* bloodstream infections in Switzerland: a nationwide surveillance study (2008–2021). Infection 2023; 51: 1025–31. 10.1007/s15010-023-01980-6 36732413 PMC10352440

[4] Gagliotti C, Högberg LD, Billström H et al *Staphylococcus aureus* bloodstream infections: diverging trends of meticillin-resistant and meticillin-susceptible isolates, EU/EEA, 2005 to 2018. Euro Surveill 2021; 26: 2002094. 10.2807/1560-7917.Es.2021.26.46.2002094 34794536 PMC8603406

[5] Gudiol F, Aguado JM, Almirante B et al Diagnosis and treatment of bacteremia and endocarditis due to *Staphylococcus aureus*. A clinical guideline from the Spanish Society of Clinical Microbiology and Infectious Diseases (SEIMC). Enferm Infecc Microbiol Clin 2015; 33: 625.e1–e23. 10.1016/j.eimc.2015.03.015 25937457

[6] Liu C, Bayer A, Cosgrove SE et al Clinical practice guidelines by the Infectious Diseases Society of America for the treatment of methicillin-resistant *Staphylococcus aureus* infections in adults and children. Clin Infect Dis 2011; 52: e18–55. 10.1093/cid/ciq146 21208910

[7] Madhavan T, Quinn EL, Freimer E et al Clinical studies of cefazolin and comparison with other cephalosporins. Antimicrob Agents Chemother 1973; 4: 525–31. 10.1128/aac.4.5.525 4791486 PMC444589

[8] Weis S, Kesselmeier M, Davis JS et al Cefazolin versus anti-staphylococcal penicillins for the treatment of patients with *Staphylococcus aureus* bacteraemia. Clin Microbiol Infect 2019; 25: 818–27. 10.1016/j.cmi.2019.03.010 30928559

[9] Rindone JP, Mellen CK. Meta-analysis of trials comparing cefazolin to antistaphylococcal penicillins in the treatment of methicillin-sensitive *Staphylococcus aureus* bacteraemia. Br J Clin Pharmacol 2018; 84: 1258–66. 10.1111/bcp.13554 29600576 PMC5980628

[10] Youngster I, Shenoy ES, Hooper DC et al Comparative evaluation of the tolerability of cefazolin and nafcillin for treatment of methicillin-susceptible *Staphylococcus aureus* infections in the outpatient setting. Clin Infect Dis 2014; 59: 369–75. 10.1093/cid/ciu301 24785233 PMC4110443

[11] Bard D, Hindler J, Gold JA et al Rationale for eliminating *Staphylococcus* breakpoints for β-lactam agents other than penicillin, oxacillin or cefoxitin, and ceftaroline. Clin Infect Dis 2014; 58: 1287–96. 10.1093/cid/ciu043 24457339 PMC5734619

[12] Farrell DJ, Castanheira M, Mendes RE et al *In vitro* activity of ceftaroline against multidrug-resistant *Staphylococcus aureus* and *Streptococcus pneumoniae*: a review of published studies and the AWARE surveillance program (2008–2010). Clin Infect Dis 2012; 55: S206–14. 10.1093/cid/cis563 22903953

[13] Kang N, Housman ST, Nicolau DP. Assessing the surrogate susceptibility of oxacillin and cefoxitin for commonly utilized parenteral agents against methicillin-susceptible *Staphylococcus aureus*: focus on ceftriaxone discordance between predictive susceptibility and *in vivo* exposures. Pathogens 2015; 4: 599–605. 10.3390/pathogens4030599 26264030 PMC4584275

[14] CLSI. *Performance Standards for Antimicrobial Susceptibility Testing—Thirty-Fourth Edition: M100*. CLSI, 2024.

[15] CLSI. *Methods for Dilution Antimicrobial Susceptibility Tests for Bacteria That Grow Aerobically: M07*. CLSI, 2024.

[16] CLSI. *Performance Standards for Antimicrobial Susceptibility Testing—Twenty-Second Informational Supplement: M100-S22*. CLSI, 2012.

[17] Enright MC, Day NP, Davies CE et al Multilocus sequence typing for characterization of methicillin-resistant and methicillin-susceptible clones of *Staphylococcus aureus*. J Clin Microbiol 2000; 38: 1008–15. 10.1128/jcm.38.3.1008-1015.2000 10698988 PMC86325

[18] CLSI. *Performance Standards for Antimicrobial Susceptibility Testing—Twenty-Third Informational Supplement: M100-S23*. CLSI, 2013.

[19] Reichmann NT, Pinho MG. Role of SCC*mec* type in resistance to the synergistic activity of oxacillin and cefoxitin in MRSA. Sci Rep 2017; 7: 6154. 10.1038/s41598-017-06329-2 28733674 PMC5522475

[20] Memmi G, Filipe SR, Pinho MG et al *Staphylococcus aureus* PBP4 is essential for beta-lactam resistance in community-acquired methicillin-resistant strains. Antimicrob Agents Chemother 2008; 52: 3955–66. 10.1128/aac.00049-08 18725435 PMC2573147

[21] Kosowska-Shick K, McGhee PL, Appelbaum PC. Affinity of ceftaroline and other beta-lactams for penicillin-binding proteins from *Staphylococcus aureus* and *Streptococcus pneumoniae*. Antimicrob Agents Chemother 2010; 54: 1670–7. 10.1128/aac.00019-10 20194704 PMC2863635

[22] Fontana R, Cornaglia G, Ligozzi M et al The final goal: penicillin-binding proteins and the target of cephalosporins. Clin Microbiol Infect 2000; 6(Suppl 3): 34–40. 10.1111/j.1469-0691.2000.tb02038.x 11449647

[23] Lee SO, Lee S, Park S et al *In vivo* evaluation of cefazolin inoculum effect in the treatment of experimental *Staphylococcus aureus* pneumonia with cefazolin. J Antimicrob Chemother 2025; 80: 1287–90. 10.1093/jac/dkaf065 40037650

[24] Nannini EC, Singh KV, Arias CA et al *In vivo* effects of cefazolin, daptomycin, and nafcillin in experimental endocarditis with a methicillin-susceptible *Staphylococcus aureus* strain showing an inoculum effect against cefazolin. Antimicrob Agents Chemother 2013; 57: 4276–81. 10.1128/aac.00856-13 23796934 PMC3754321

[25] Dingle TC, Gamage D, Gomez-Villegas S et al Prevalence and characterization of the cefazolin inoculum effect in North American methicillin-susceptible *Staphylococcus aureus* isolates. J Clin Microbiol 2022; 60: e0249521. 10.1128/jcm.02495-21 35578988 PMC9297818

[26] Winans SA, Luce AM, Hasbun R. Outpatient parenteral antimicrobial therapy for the treatment of methicillin-susceptible *Staphylococcus aureus*: a comparison of cefazolin and ceftriaxone. Infection 2013; 41: 769–74. 10.1007/s15010-013-0477-0 23686435

[27] Paul M, Zemer-Wassercug N, Talker O et al Are all beta-lactams similarly effective in the treatment of methicillin-sensitive *Staphylococcus aureus* bacteraemia? Clin Microbiol Infect 2011; 17: 1581–6. 10.1111/j.1469-0691.2010.03425.x 21073629

[28] Heffernan AJ, Sime FB, Lim SMS et al Pharmacodynamics of ceftriaxone for the treatment of methicillin-susceptible *Staphylococcus aureus*: is it a viable treatment option? Int J Antimicrob Agents 2022; 59: 106537. 10.1016/j.ijantimicag.2022.106537 35093539

[29] Zelenitsky SA, Beahm NP, Iacovides H et al Limitations of ceftriaxone compared with cefazolin against MSSA: an integrated pharmacodynamic analysis. J Antimicrob Chemother 2018; 73: 1888–94. 10.1093/jac/dky120 29635472

[30] Nguyen HM, Jones RN. Treatment of methicillin-susceptible *Staphylococcus aureus* osteoarticular and prosthetic joint infections: using the oxacillin minimum inhibitory concentration to guide appropriate ceftriaxone use. Clin Infect Dis 2013; 57: 161–2. 10.1093/cid/cit188 23537905

[31] Sader HS, Carvalhaes CG, Mendes RE. Ceftaroline activity against *Staphylococcus aureus* isolated from patients with infective endocarditis, worldwide (2010–2019). Int J Infect Dis 2021; 102: 524–8. 10.1016/j.ijid.2020.11.130 33207274

[32] Biedenbach DJ, Alm RA, Lahiri SD et al *In vitro* activity of ceftaroline against *Staphylococcus aureus* isolated in 2012 from Asia-Pacific countries as part of the AWARE surveillance program. Antimicrob Agents Chemother 2016; 60: 343–7. 10.1128/aac.01867-15 26503659 PMC4704164

[33] Ho CM, Hsueh PR, Liu CY et al Prevalence and accessory gene regulator (*agr*) analysis of vancomycin-intermediate *Staphylococcus aureus* among methicillin-resistant isolates in Taiwan–SMART program, 2003. Eur J Clin Microbiol Infect Dis 2010; 29: 383–9. 10.1007/s10096-009-0868-4 20155296

[34] Ho CM, Ho MW, Lee CY et al Clonal spreading of methicillin-resistant SCC*mec Staphylococcus aureus* with specific *spa* and *dru* types in central Taiwan. Eur J Clin Microbiol Infect Dis 2012; 31: 499–504. 10.1007/s10096-011-1338-3 21789606

[35] Chen YJ, Chen PA, Chen CJ et al Molecular characteristics and clinical features of pediatric methicillin-susceptible *Staphylococcus aureus* infection in a medical center in northern Taiwan. BMC Infect Dis 2019; 19: 402. 10.1186/s12879-019-4033-0 31077140 PMC6509804

[36] Chen FJ, Siu LK, Lin JC et al Molecular typing and characterization of nasal carriage and community-onset infection methicillin-susceptible *Staphylococcus aureus* isolates in two Taiwan medical centers. BMC Infect Dis 2012; 12: 343. 10.1186/1471-2334-12-343 23228040 PMC3522061

[37] Goering RV, Shawar RM, Scangarella NE et al Molecular epidemiology of methicillin-resistant and methicillin-susceptible *Staphylococcus aureus* isolates from global clinical trials. J Clin Microbiol 2008; 46: 2842–7. 10.1128/jcm.00521-08 18614654 PMC2546764

