# Supplementary material for: Re-examining cephalosporin activity against methicillin-susceptible Staphylococcus aureus among clinical isolates from southern Taiwan
Source: JAC Antimicrob Resist. 2026 Mar 5;8(1):dlag029. doi: 10.1093/jacamr/dlag029 (PMC12961745; doi:10.1093/jacamr/dlag029)
Supplement: dlag029_Supplementary_Data [file dlag029_supplementary_data.zip › Supplementary_data_0211_CLEAN.docx]

**Table S1. Quality control minimum inhibitory concentration results for *Staphylococcus aureus* ATCC 29213**

| **Antimicrobial agent** | **CLSI QC MIC range (mg/L)** | **Observed MIC range (mg/L)** | **No. of QC runs** |
| --- | --- | --- | --- |
| Oxacillin | 0.12-0.5 | 0.25-0.5 | 25 |
| Cefazolin | 0.25-1 | 0.5-1 | 25 |
| Cefuroxime | 0.5-2 | 1-2 | 25 |
| Ceftriaxone | 1-8 | 2–4 | 25 |
| Cefepime | 1-4 | 1-2 | 25 |
| Ceftaroline | 0.12–0.5 | 0.25–0.5 | 25 |

QC: quality control; MIC: minimum inhibitory concentration

**Table S2. Cephalosporin minimum inhibitory concentration breakpoints for *Staphylococcus aureus*.**

| **Antimicrobial agents** | **MIC Breakpoint (mg/L)** | | | **Reference** |
| --- | --- | --- | --- | --- |
|  | **S** | **I /SDD** | **R** |  |
| Cefazolin | ≤8 | 16 | ≥32 | CLSI 2012 M100-S22 |
| Cefuroxime | ≤8 | 16 | ≥32 |  |
| Ceftriaxone | ≤8 | 16-32 | ≥64 |  |
| Cefepime | ≤8 | 16 | ≥32 |  |
| Ceftaroline | ≤1 | 2-4* | ≥8 | CLSI 2024 M100-Ed34 |
| Oxacillin | ≤2 |  | ≥4 | CLSI 2024 M100-Ed34 |

I: intermediate; MIC: minimum inhibitory concentration; R: resistant; S: susceptible; SDD: susceptible-dose dependent

* Susceptible-dose dependent for ceftaroline

**Table S3. Clonal complex assignments based on multilocus sequence typing for ceftaroline-resistant or susceptible-dose dependent methicillin-susceptible *Staphylococcus aureus* isolates.**

| **Clonal complex** | **Sequence type** |
| --- | --- |
| CC7210 | ST7210, ST9790, ST9796, ST9798 |
| CC8923 | ST8923, ST9792 |
| CC8928 | ST8928, ST9793, ST9794 |
| CC9787 | ST9787, ST9785 |
| CC9791 | ST9791, ST9789 |
| CC9802 | ST9802, ST8838, ST9716, ST9797 |
| CC9808 | ST9808, ST8988, ST9806 |

**Figure S1. Distribution of minimum inhibitory concentrations for five cephalosporins against 514 methicillin-susceptible *Staphylococcus aureus* isolates.**

The histograms display the frequency distribution of minimum inhibitory concentration (MICs; mg/L) for cefazolin, cefuroxime, ceftriaxone, cefepime, and ceftaroline. Blue (striped) bars indicate susceptible isolates; grey (solid) bars indicate intermediate or susceptible-dose dependent (SDD) isolates; and orange (patterned) bars indicate resistant isolates.

**ALT TEXT (figure S1):** Five horizontal bar charts arranged in a grid showing the minimum inhibitory concentration (MIC) distributions for cefazolin, cefuroxime, ceftriaxone, cefepime, and ceftaroline against 514 MSSA isolates. On each chart, the Y-axis lists MIC values (mg/L) and the X-axis shows the number of isolates.

**Figure S2. Temporal and regional comparison of cephalosporin non-susceptibility among** **methicillin-susceptible *Staphylococcus aureus* isolates.**

The grouped bar chart illustrates non-susceptibility rates for five cephalosporins stratified by collection period and hospital. At Chiayi Chang Gung Memorial Hospital (CCGMH), a significant decline in non-susceptibility was observed between the earlier (2015–2019, n = 186) and later (2020–2024, n = 160) periods for cefazolin, cefuroxime, ceftriaxone, and cefepime (*p* < 0.01). In contrast, ceftaroline resistance rate remained consistently low with no significant temporal variation. In the recent period (2020–2024), susceptibility profiles were highly comparable between CCGMH (n = 160) and Kaohsiung Chang Gung Memorial Hospital (KCGMH, n = 168), with no statistically significant differences observed.

**ALT TEXT (figure S2):** Grouped bar chart with five cephalosporins on the x-axis and non-susceptibility percentage on the y-axis, comparing temporal and regional cohorts. Each drug displays clustered bars for CCGMH 2015–2019 (n=186), CCGMH 2020–2024 (n=160), and KCGMH 2020–2024 (n=168), with significance markers indicating statistical differences between the two temporal periods at CCGMH.

**Figure S3. Percentage of methicillin-susceptible *Staphylococcus aureus* isolates non-susceptible to cephalosporins, stratified by oxacillin minimum inhibitory concentration <0.25 mg/L and ≥0.25 mg/L.**

Non-susceptibility to first- through fourth-generation cephalosporins (cefazolin, cefuroxime, ceftriaxone, and cefepime) remained uncommon when the oxacillin minimum inhibitory concentration (MIC) was <0.25 mg/L (1.0%–1.3%) but increased substantially at MICs ≥0.25 mg/L (16.5%–23.1%), indicating that higher oxacillin MICs are broadly associated with elevated cephalosporin MICs. In contrast, the increase in ceftaroline non-susceptibility was less pronounced, rising from 0.5% to 4.1% across the same oxacillin MIC strata.

**ALT TEXT (figure S3):** Grouped bar chart displaying the percentage of *Staphylococcus aureus* isolates non-susceptible to five cephalosporins (cefazolin, cefuroxime, ceftriaxone, cefepime, and ceftaroline), stratified by oxacillin minimum inhibitory concentration (MIC) <0.25 mg/L and ≥0.25 mg/L. For each antibiotic, paired patterned bars illustrate changes in non-susceptibility across oxacillin MIC strata, with a pronounced increase observed for all agents except ceftaroline at higher oxacillin MICs.

**Figure S4. Bubble charts illustrating the correlation between oxacillin minimum inhibitory concentrations and cephalosporin MICs among 514 methicillin-susceptible *Staphylococcus aureus* isolates.**

Panels show the minimum inhibitory concentration (MIC) distributions for (A) cefazolin, (B) cefuroxime, (C) ceftriaxone, (D) cefepime, and (E) ceftaroline plotted against corresponding oxacillin MICs. Each bubble represents the number of isolates at a given MIC combination, with bubble size proportional to isolate count. Blue bubbles denote isolates classified as susceptible, whereas orange bubbles represent isolates classified as intermediate, resistant, or susceptible-dose dependent for ceftaroline.

**ALT TEXT (figure S4):** A composite figure consisting of five bubble charts illustrating the correlation between oxacillin minimum inhibitory concentrations (MICs) (y-axis) and the MICs of five cephalosporins (x-axis): cefazolin, cefuroxime, ceftriaxone, cefepime, and ceftaroline. In each chart, the bubble size is proportional to the number of bacterial isolates at that specific MIC combination.
