# Supplementary figures and images for: Re-examining cephalosporin activity against methicillin-susceptible Staphylococcus aureus among clinical isolates from southern Taiwan
Source: JAC Antimicrob Resist. 2026 Mar 5;8(1):dlag029. doi: 10.1093/jacamr/dlag029 (PMC12961745; doi:10.1093/jacamr/dlag029)

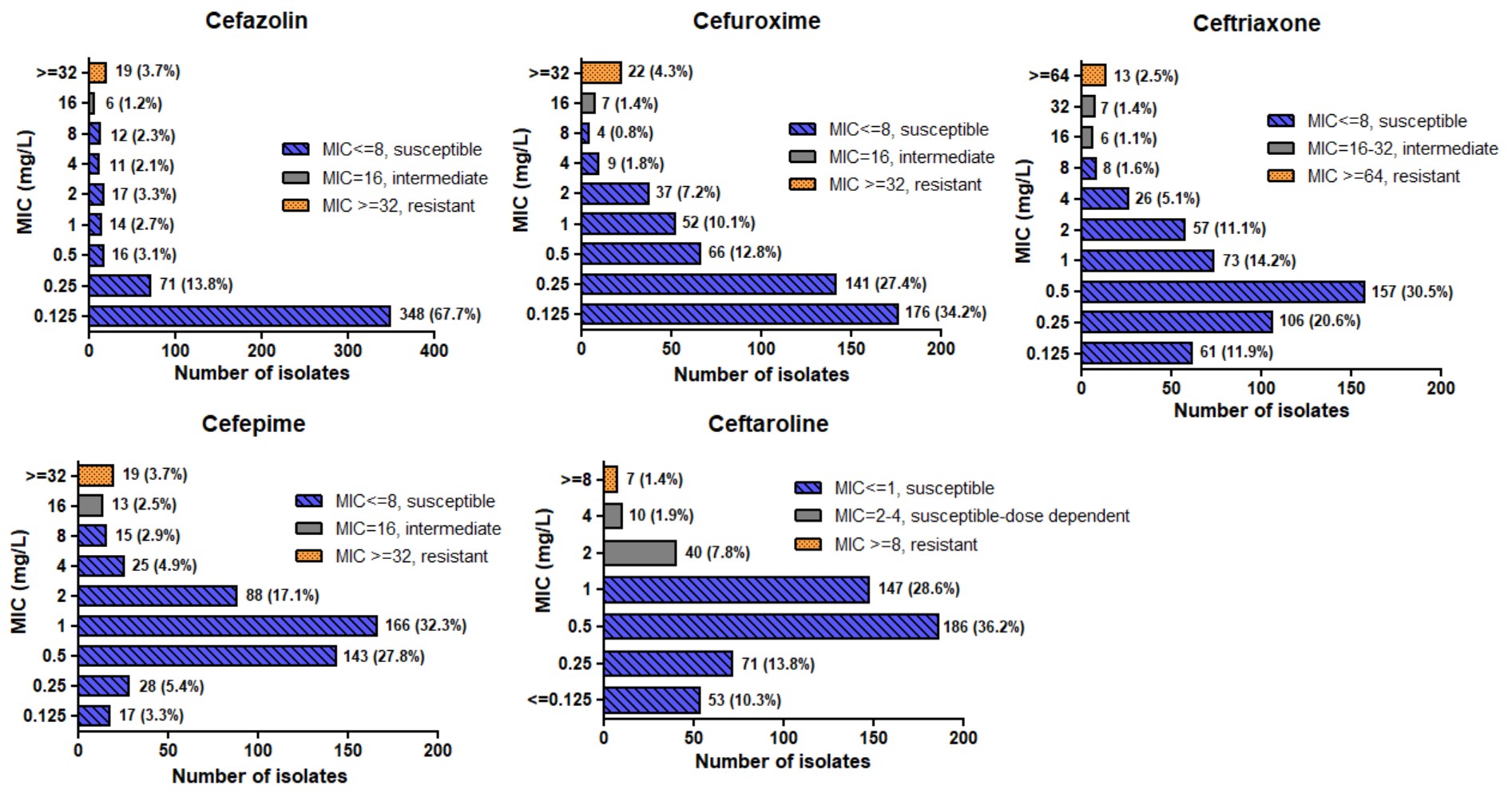

Supplement: dlag029_Supplementary_Data [file dlag029_supplementary_data.zip › Figure S1.jpg]

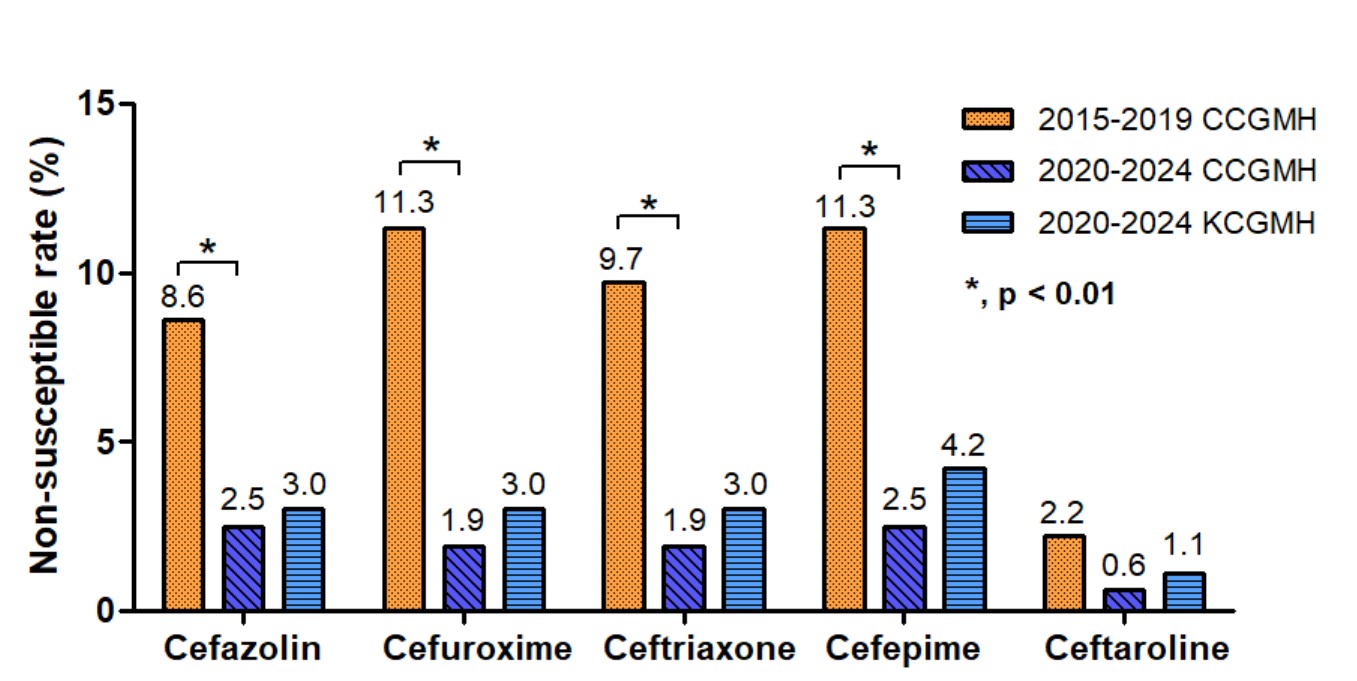

Supplement: dlag029_Supplementary_Data [file dlag029_supplementary_data.zip › Figure S2.jpg]

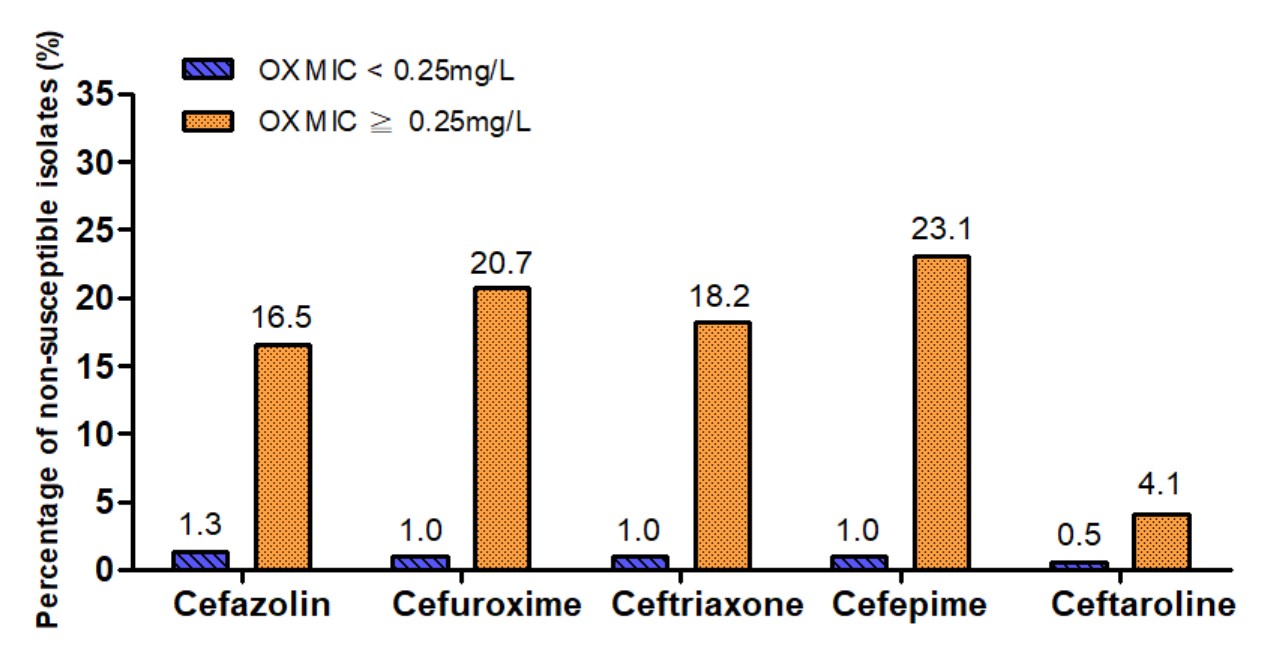

Supplement: dlag029_Supplementary_Data [file dlag029_supplementary_data.zip › Figure S3.jpg]

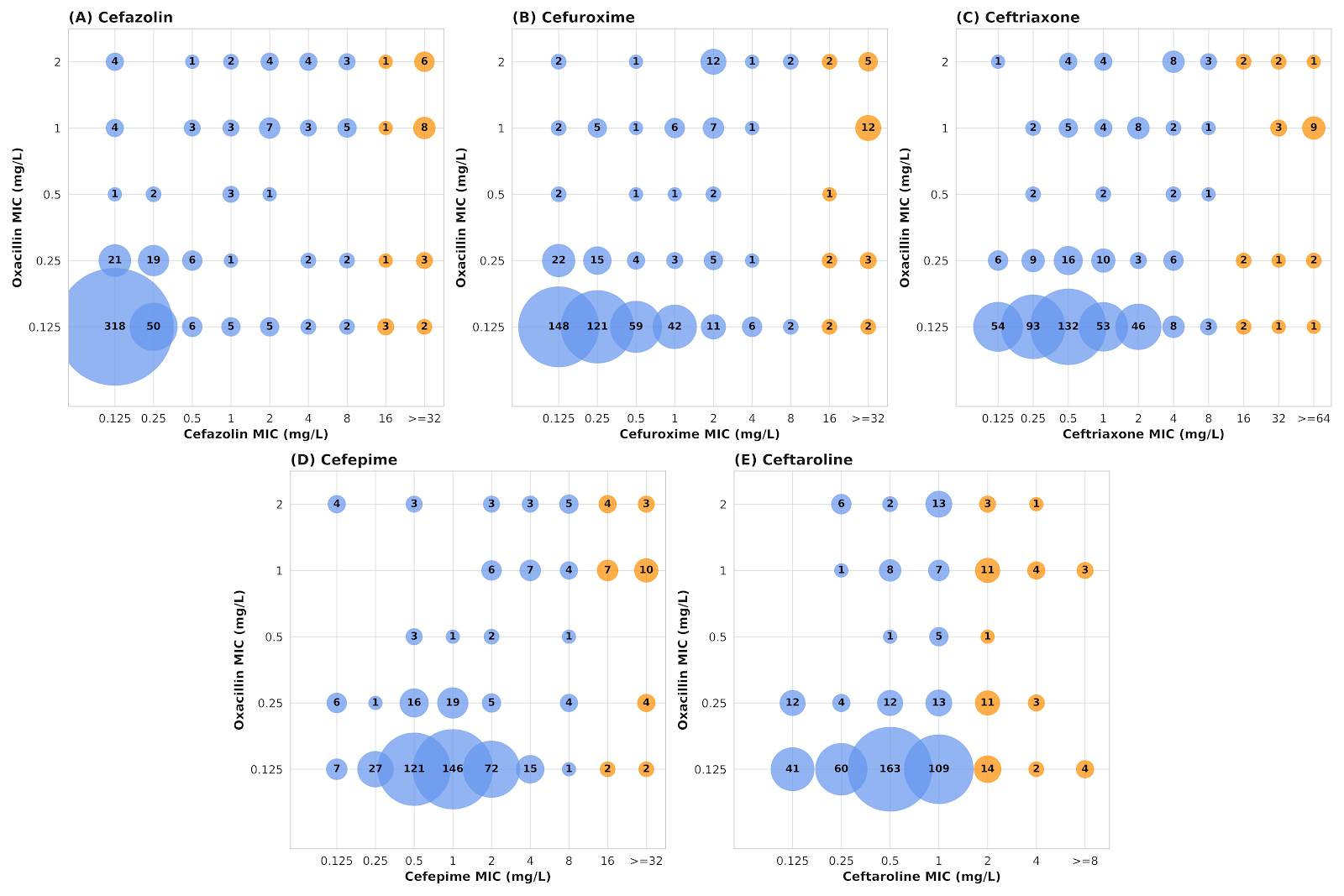

Supplement: dlag029_Supplementary_Data [file dlag029_supplementary_data.zip › Figure S4.png]
